# Supplementary material for: Late Miocene climate cooling and intensification of southeast Asian winter monsoon
Source: Nat Commun. 2018 Apr 20;9:1584. doi: 10.1038/s41467-018-03950-1 (PMC5910391; doi:10.1038/s41467-018-03950-1)
Supplement: Supplementary file 1 — Supplementary Information [file 41467_2018_3950_MOESM1_ESM.pdf]

Late Miocene climate cooling and intensification of southeast Asian winter monsoon

Holbourn et al.

Supplementary Notes 1–4, Supplementary Figures 1–10, Supplementary Tables 1–2 and Supplementary References

---

Supplementary Note 1: Site 1146 benthic and planktic stable isotope records and astronomically-tuned isotope stratigraphy between 9 and 5 Ma

We analyzed 2230 samples for benthic foraminiferal isotopes and 2249 samples for planktonic foraminiferal isotopes along a revised composite sediment sequence from Holes 1146A and 1146C. The average sample spacing and temporal resolution of the data sets are 3.7 cm and 1.8 kyr, respectively (Supplementary Fig. 1A–B). Temporal trends in planktic and benthic foraminiferal $\delta^{18}O$ are shown in Supplementary Fig. 2. To derive an astronomically-tuned age model over the interval 8.6 to 5.0 Ma, we used an equal mixture of eccentricity and tilt (ET) from ref. 1 as a tuning target (Supplementary Figs. 3–4). We did not include a precession component in the tuning target to remain independent of possible changes in the phase relationship of deep water oxygen isotopes to northern/southern hemisphere precessional insolation. Below 8.6 Ma, we used tie points to ET defined in ref. 2. Over this interval, we optimized tuning by reducing the number of tie points without changing the original age model in ref. 2. Between 6 and 5 Ma, prominent glaciation/deep water cooling events (benthic $\delta^{18}O$ maxima) T8, TG4, TG12, TG18 TG20, TG22 and TG32 form the backbone of the oxygen isotope stratigraphy (e.g., refs. 3–4). Age correlation points used to derive astronomically-tuned age model over interval 9–5 Ma are provided in Supplementary Table 1A. Astronomically-tuned ages of $\delta^{18}O$ maxima (T and TG events) are provided in Supplementary Table 1B, based

on correlation to ET or ET-precession (P) minima (without phasing). Note that deviation of ages derived using these two tuning targets is on average 4 kyr with a maximum of 8 kyr and a minimum of 1 kyr.

Supplementary Table 1A. Age correlation points used to derive astronomically-tuned age model over interval 9–5 Ma.

**Depth (Rmcd*)**	**Age (Ma)**
299.70	5.120
301.28	5.178
303.48	5.259
311.78	5.549
313.53	5.590
316.48	5.709
317.92	5.751
319.28	5.793
325.08	5.997
325.58	6.017
327.13	6.053
328.08	6.121
334.01	6.304
345.33	6.675
348.48	6.793
356.18	7.044
357.58	7.080
359.73	7.162
361.28	7.243
363.58	7.324
365.43	7.362
369.43	7.481
370.83	7.525
373.43	7.645
381.23	7.890
384.28	8.010
392.28	8.266
395.33	8.384
398.63	8.462
402.24	8.545
403.94	8.612
405.42	8.663
410.17	8.868
411.67	8.953
413.82	9.038
415.67	9.125
416.62	9.157
418.22	9.241
421.72	9.403
424.83	9.521
430.52	9.763
434.02	9.883

*Rmcd: revised meter composite depth

Supplementary Table 1B. Comparison of astronomically-tuned ages of benthic $\delta^{18}O$ maxima T8, TG4, TG12, TG18 TG20, TG22 and TG32 using either ET or ET-P from ref. 1 as tuning target.

**Event**	**Rmcd***	**Benthic $\delta^{18}O$**	**ET**	**ET-P**
T8	301.28 m	2.81 ‰	5.178	5.172
TG4	mid point between			
	303.48 and 303.73 m	2.73–2.71 ‰	5.259	5.265
TG12	311.78 m	3.04 ‰	5.549	5.550
TG14	mid point between			
	313.53 and 313.78 m	2.89–2.89 ‰	5.590	5.589
TG18	316.48 m	2.72 ‰	5.709	5.701
TG20	317.92 m	3.10 ‰	5.751	5.753
TG22	319.28 m	2.89 ‰	5.793	5.794
TG32	325.08 m	2.88 ‰	5.997	6.002

*Rmcd: revised meter composite depth.

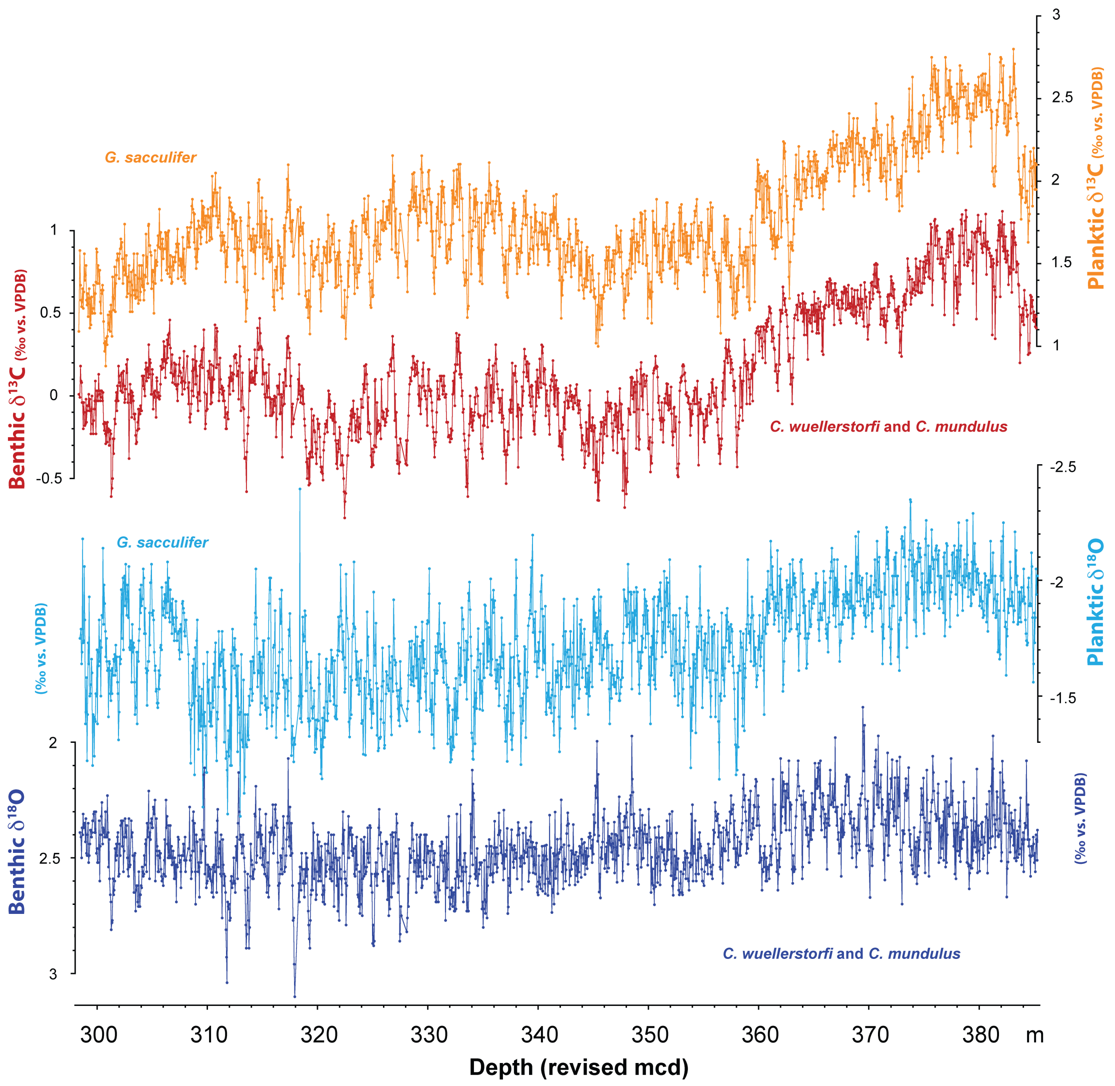

Supplementary Fig. 1A. Benthic *(Cibicidoides wuellerstorfi*, *Cibicidoides mundulus)* and planktic (*Globigerinoides sacculifer*) δ^{18}O and δ^{13}C at ODP Site 1146, plotted against depth (revised mcd). Original data.

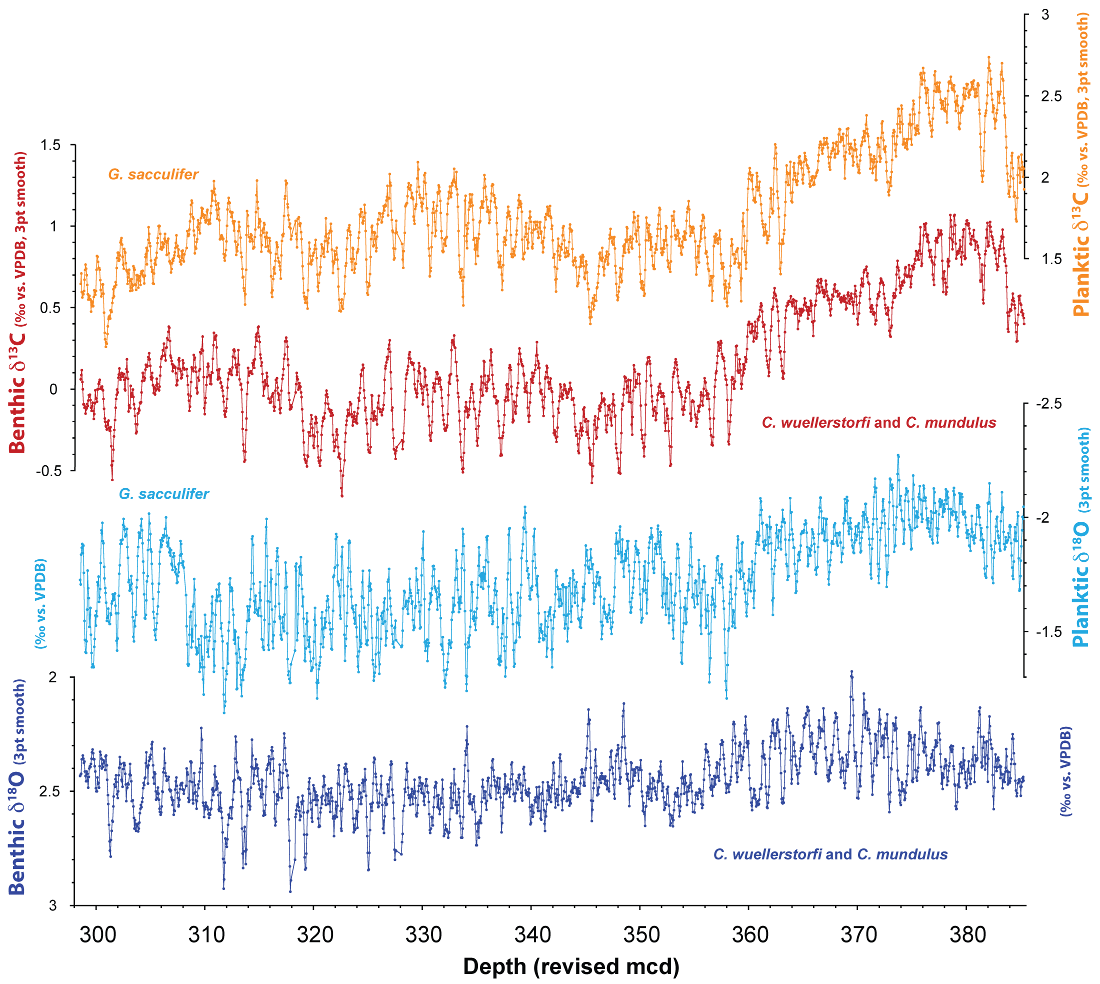

Supplementary Fig. 1B. Benthic *(Cibicidoides wuellerstorfi*, *Cibicidoides mundulus)* and planktic (*Globigerinoides sacculifer*) δ^{18}O and δ^{13}C at ODP Site 1146, plotted against depth (revised mcd). 3 pt smooth: 3 point moving mean.

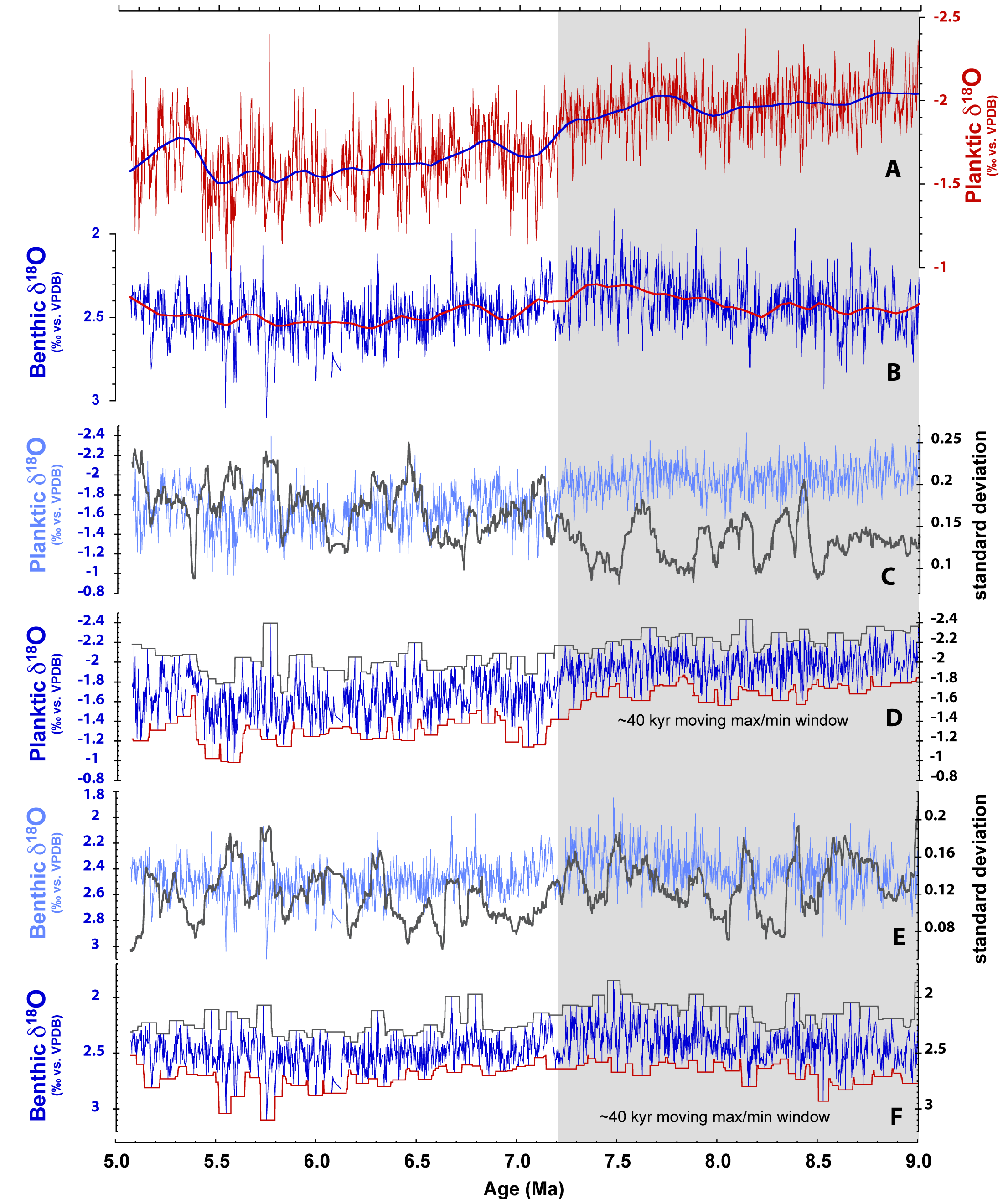

Supplementary Fig. 2. A–B: Temporal evolution of planktic and benthic foraminiferal $\delta^{18}O$ at ODP Site 1146 over interval 9–5 Ma. Evaluation of long-term trends in planktic (**A**) and benthic (**B**) $\delta^{18}O$. Smooth curves in A and B fitted using locally weighted least squared error (Lowess) method. **C:** Planktic $\delta^{18}O$ with 50 point moving window standard deviation. **D:** Amplitude variance in planktic $\delta^{18}O$ (40 kyr moving window). **E:** Benthic $\delta^{18}O$ with 50 point moving window standard deviation. **F:** Amplitude variance in benthic $\delta^{18}O$ (40 kyr moving window).

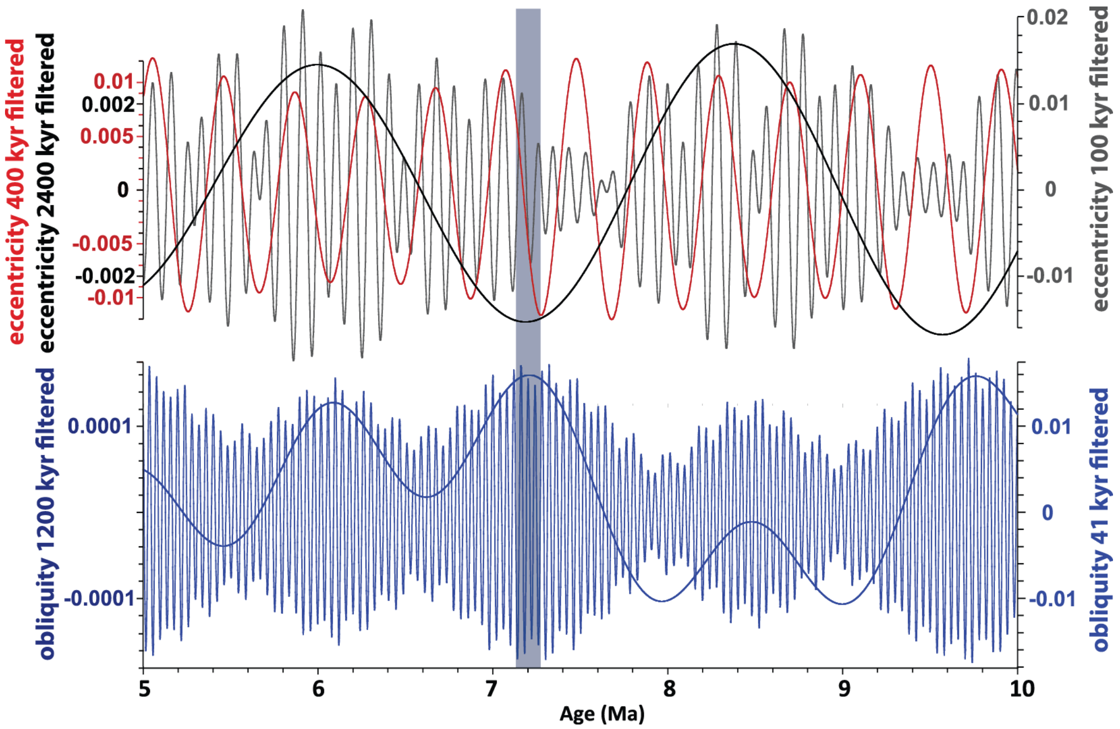

Supplementary Fig. 3. Astronomical parameters from ref. 1 over interval 10–5 Ma. Data filtered with AnalySeries[5] using Gaussian filters centered at wavelenghts of 41 kyr (0.2433 frequency, 0.007 bandwidth), 100 kyr (0.01 frequency, 0.004 bandwidth), 400 kyr (0.0025 frequency, 0.0005 bandwidth), 1.2 Myr (0.00083 frequency, 0.0001 bandwidth) and 2.4 Myr (0.0004166 frequency, 0.0001 bandwidth). Note that ~80 kyr long positive benthic $\delta^{18}O$ excursion (gray band) at ~7.2-7.0 Ma coincides with minima in eccentricity (100 kyr, 400 kyr and 2.4 Myr amplitude modulation).

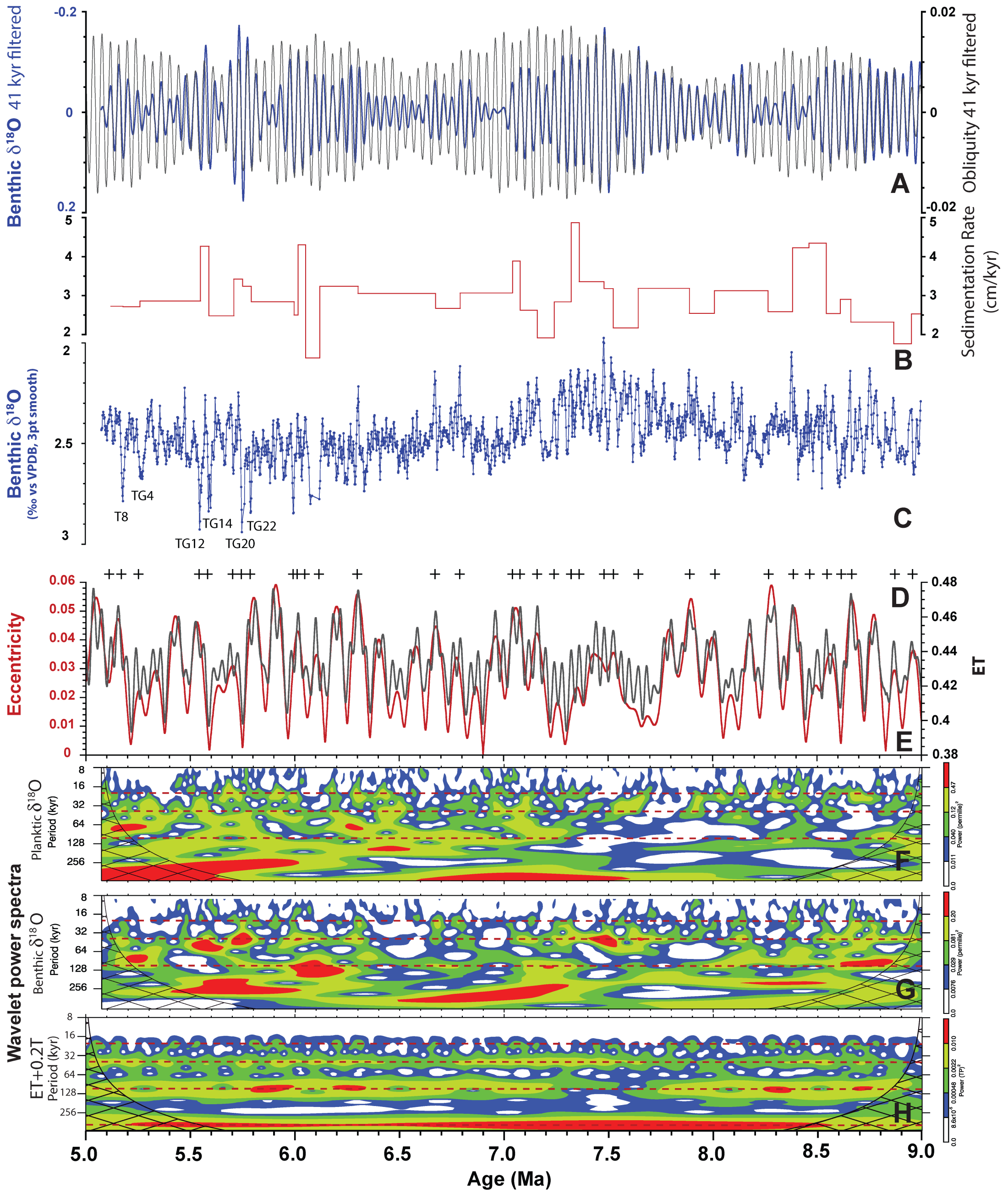

Supplementary Fig. 4. Astronomical tuning of high-resolution (~2 kyr) benthic δ^{18}O at ODP Site 1146 over interval 9–5 Ma. **A:** Comparison of 41 kyr filtered benthic δ^{18}O and obliquity (Gaussian bandpass filter with bandwidth of 0.004 centered at frequency of 0.0243). **B:** Sedimentation rates. **C:** Benthic foraminiferal δ^{18}O; 3 pt smooth: 3 pt moving mean. **D:** Tie points marked by gray crosses. **E:** Eccentricity plus obliquity (ET) from ref. 1. **F:** Wavelet power spectrum of planktic δ^{18}O using Morlet wavelet with six parameters (as detailed in ref. 6), scale width of 0.01, and start scale of 2, contour levels represent more than 75% (red), 50% (yellow), 25% (green), and 5% (blue) of wavelet power. **G:** Wavelet power spectrum of benthic δ^{18}O (settings are the same as in F). **H:** Wavelet power spectrum of ET+0.2P (settings are the same as in F).

Supplementary Note 2: Coherence and phase relationship between planktic and benthic δ^{13}C and δ^{18}O

Blackman-Tukey power spectra of benthic and planktic δ^{18}O time series indicate dominant response of benthic δ^{18}O to 41 kyr obliquity and stronger response of mixed layer δ^{18}O to 23 kyr precession (Supplementary Fig. 5). Cross wavelet coherency and phase analyses indicate that benthic and planktic δ^{13}C are strongly coherent and in phase at the obliquity and eccentricity (100 and 400 kyr) bands, whereas benthic and planktic δ^{18}O exhibit relatively low coherence and variable phase relationship at all main orbital bands (Supplementary Fig. 6).

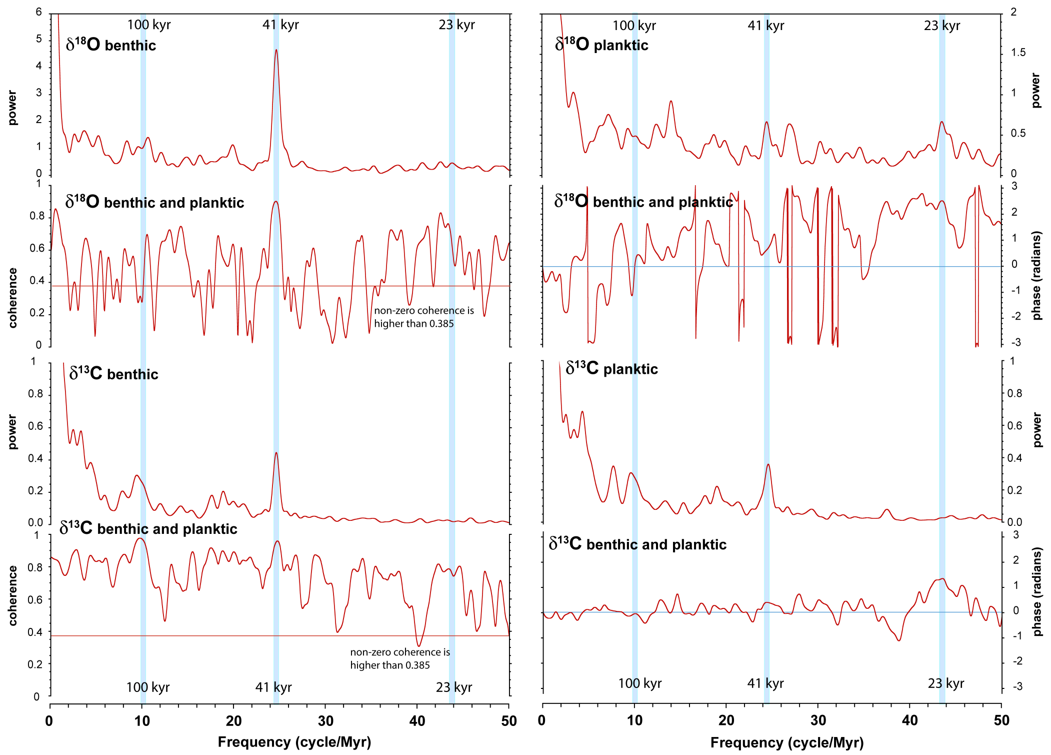

Supplementary Fig. 5. Benthic and planktic δ^{18}O and δ^{13}C power spectra, coherence and phase relationship over interval 9–5 Ma. Blackman-Tukey cross spectra using a Bartlett window with pre-whitening and linear trend removed. Bandwidth is 1.273, non zero coherence is higher than 0.385, error estimation on the power spectrum is $0.6255 < \Delta Power/Power < 2.055$. Positive values in the phase diagrams indicate lead of benthic δ^{18}O and δ^{13}C over planktic δ^{18}O and δ^{13}C.

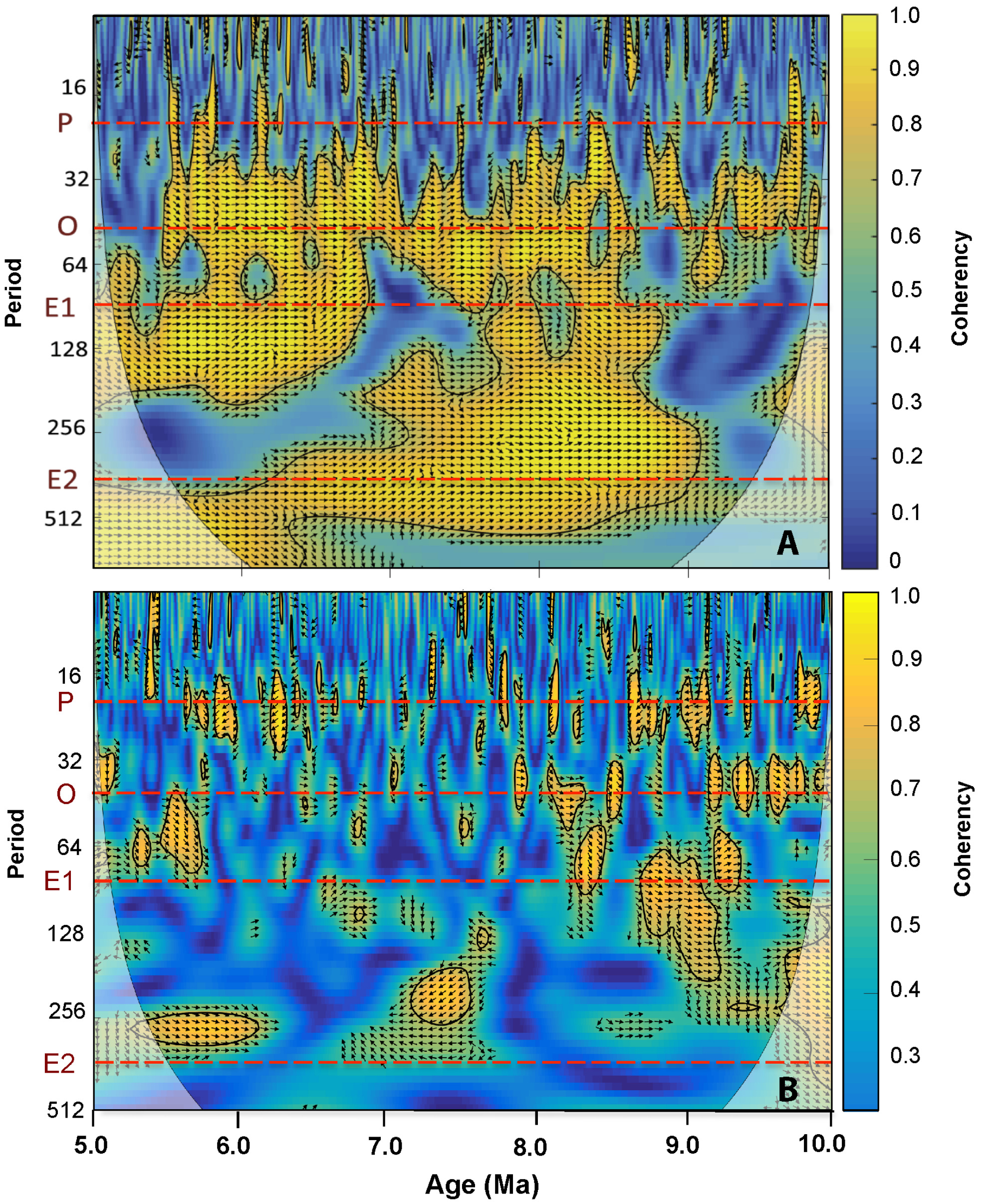

Supplementary Fig. 6. Cross wavelet coherency and phase between planktic and benthic foraminiferal $\delta^{13}C$ and $\delta^{18}O$ over interval 10–5 Ma at Site 1146. **A:** Cross wavelet coherency and phase between planktic and benthic foraminiferal $\delta^{13}C$. Black contours around areas of high coherence (yellow shades) demarcate 5 % significance level against red noise. Vectors point right when benthic $\delta^{13}C$ is in-phase with planktic $\delta^{13}C$, left when benthic $\delta^{13}C$ is anti-phase with planktic $\delta^{13}C$, down when benthic $\delta^{13}C$ lags planktic $\delta^{13}C$ and up when benthic $\delta^{13}C$ leads planktic $\delta^{13}C$. Wavelet Coherence Matlab Toolbox software from ref. 7. P: 23 kyr precession; O: 41 kyr obliquity; E1: 100 kyr eccentricity; E2: 400 kyr eccentricity. **B:** Cross wavelet coherency and phase between planktic and benthic foraminiferal $\delta^{18}O$. Black contours demarcate 5 % significance level against red noise. Vectors point right when benthic $\delta^{18}O$ is in-phase with planktic $\delta^{18}O$, left when benthic $\delta^{18}O$ is anti-phase with planktic $\delta^{18}O$, down when benthic $\delta^{18}O$ lags planktic $\delta^{18}O$ and up when benthic $\delta^{18}O$ leads planktic $\delta^{18}O$. Wavelet Coherence Matlab Toolbox software from ref. 7. P: 23 kyr precession; O: 41 kyr obliquity; E1: 100 kyr eccentricity; E2: 400 kyr eccentricity.

Supplementary Note 3: Mixed layer Mg/Ca temperature and $\delta^{18}O_{sw}$ reconstruction

Modern seasonal sea surface temperature (SST) and sea surface salinity (SSS) in the northern South China Sea

Oceanographic data for seasonal mixed layer temperature and salinities in the northern South China Sea are available from the World Ocean Atlas (WOA), the IFREMER/LOS Mixed Layer Depth Climatology, and the recently published SCS Physical Oceanography dataset (SCSPOD14, ref. 8). Today, the area of IODP Site 1146 is characterized by a relatively strong seasonal contrast in SST (~3°C), while seasonal differences in SSS remain small (<0.2 psu) due to the inflow of relatively saline surface water from the open West Pacific into the northern South China Sea through the Luzon (Bashi) Strait (Supplementary Fig. 7).

Miocene Mg/Ca based temperature estimates

Potential biases on Miocene foraminiferal Mg/Ca paleothermometry include: (1) uncertainties in the depth habitat of Miocene planktic foraminifers, (2) species-specific differences in Mg/Ca temperature sensitivity, (3) the influence of dissolution at low carbonate saturation state of the bottom water, (4) variability in the relationship between seawater and foraminiferal calcite Mg/Ca and (5) the lack of knowledge about the Mg/Ca of Neogene seawater.

Uncertainties in depth habitat and species-specific Mg/Ca temperature sensitivity were minimized by using an extant foraminiferal species with an established core-top calibration. The influence of dissolution at Site 1146 is low, due to the relatively shallow water depth and elevated sedimentation rates of carbonate and clay-rich sediments, resulting in rapid burial of foraminiferal tests and carbonate

saturated pore waters. The long residence times of Mg (~13 Myr) and Ca (~1.1 Myr) in the ocean[9] imply that changes in Mg/Ca of seawater should not influence the reconstruction of relative temperature changes over time intervals <1 Myr), but may impact absolute values in comparison with modern conditions. Several studies of past variability in the Mg/Ca of seawater suggested a gradual increase through the Neogene to reach modern values of ~5 mol/mol (refs. 10–15). However, huge uncertainties remain in absolute estimates[15–16].

To evaluate the potential offset of our SST estimates, we used a modified calibration equation with a late Miocene Mg/Ca of seawater in the range of 4.5 mol/mol to adjust for potential differences between Miocene and modern Mg/Ca of seawater[17]. Due to the limitations of current reconstructions of the Mg/Ca of seawater, we opted to present both corrected and uncorrected SST values in Figs. 3, 4 and 5 and Supplementary Figs. 8,9C, as our interpretations and conclusions are not primarily affected by long-term trends in the Mg/Ca of seawater. Comparison of published Uk'$_{37}$ SST records[18] to Site 1146 Mg/Ca derived mixed layer temperatures show that temperature estimates at Site 1146 are generally lower than at tropical sites (Supplementary Fig. 8A) and that the marked cooling registered in mid- and high-latitude regions between 7.2 and 5.3 Ma is also evident at Site 1146 Ma (Supplementary Fig. 8).

Regional hydrological signals in the mixed layer $\delta^{18}O_{sw}$ records

Today, mixed layer salinity at 25 m water depth is 33.9 psu during the height of the summer monsoon period in June and 34.02 psu during the dry winter monsoon season in December[19]. This seasonal variability in salinity of 0.12 psu corresponds to less than a 0.1‰ change in $\delta^{18}O_{sw}$, if we apply the $\delta^{18}O_{sw}$/salinity relationship for the

western Pacific Ocean described in ref. 20. The seasonal variability in sea surface salinity at the location of Site 1146 is also <0.2 psu (Supplementary Fig. 7). In contrast, the late Miocene 1146 $\delta^{18}O_{sw}$ variability of 1–0.5 ‰ (Supplementary Fig. 9A) would account for changes in salinity, which cannot be explained by salinity changes alone, but must involve profound changes in the local hydrological regime, including $\delta^{18}O$ of precipitation and runoff. Therefore, we do not use $\delta^{18}O_{sw}$ as a quantitative proxy of salinity or precipitation/runoff amount, but as a qualitative indicator for changes in the regional hydrological regimes between prevailing cooler/dryer (dominant winter monsoon) and warmer/wetter (tropical advection or dominant summer monsoon) conditions.

Before ~7.1 Ma, $\delta^{18}O_{sw}$ fluctuated around a mean of 0.13 ‰ with relatively low standard deviation of 0.22 ‰ (n=148, interval between 8.2 and 7.1 Ma), whereas after ~7.1 Ma, mean $\delta^{18}O$ increased to 0.35 ‰ and standard deviation increased to 0.27 ‰ (n=374, interval between 7.1 and 5.0 Ma) (Supplementary Fig. 9A). This long-term change in surface hydrology does not solely reflect decreased amount and higher variability of precipitation/runoff, but also relates to changes in provenance and seasonality. Present day patterns of precipitation hydrology in China are characterized by an extreme latitudinal gradient in seasonality and $\delta^{18}O$ of rainfall[21] with an unusual seasonality of rainwater $\delta^{18}O$ ($\delta^{18}O$ depleted summer precipitation) in the southern part of China, whereas the northern part of China is characterized by a high temperature contribution to precipitation $\delta^{18}O$ and, thus, strongly $\delta^{18}O$ depleted winter precipitation[21]. Cooling and drying of the Asian landmass and a related southward shift of the average summer position of the ITCZ in the late Miocene would drive the hydrological regime in southern China from a more tropical convective rainfall dominated system with prevailing amount contribution to $\delta^{18}O$ rainwater into a more

seasonal monsoonal hydrological system with dry winter seasons and increased temperature control on $\delta^{18}O$ rainwater during colder climate intervals. This change is reflected by a substantial increase in the mean and variance of $\delta^{18}O_{sw}$ at Site 1146 (Supplementary Fig. 9A).

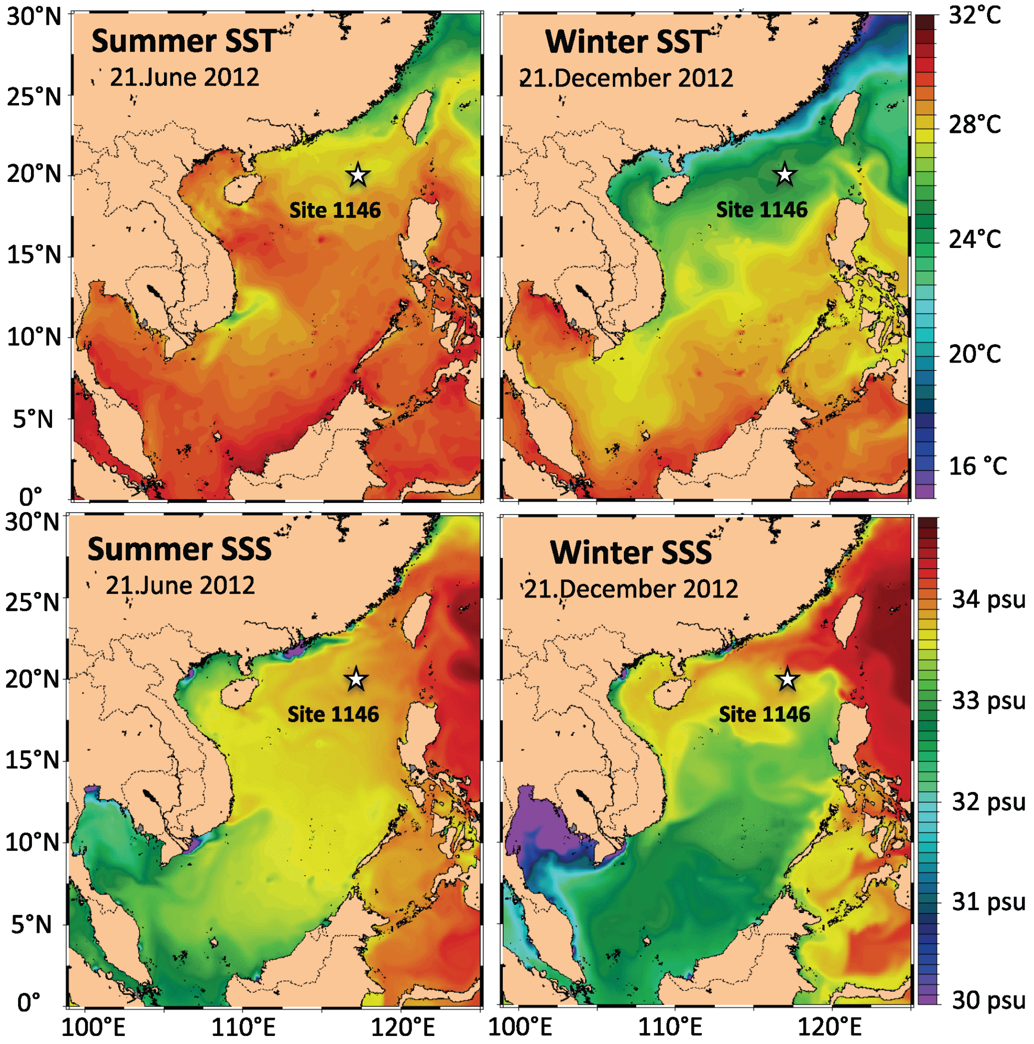

Supplementary Fig. 7. Location of ODP Site 1146 and present day summer (21 June 2012) and winter (21 December 2012) sea surface temperature (SST) and sea surface salinity (SSS) in the South China Sea. Sea surface temperature and salinity data from Naval Research Laboratory Navy Coastal Ocean Model (NCOM) 1/8° Global NCOM Nowcast South China Sea (www7320.nrlssc.navy.mil/global_ncom/glb8_3b/html/SCS.html).

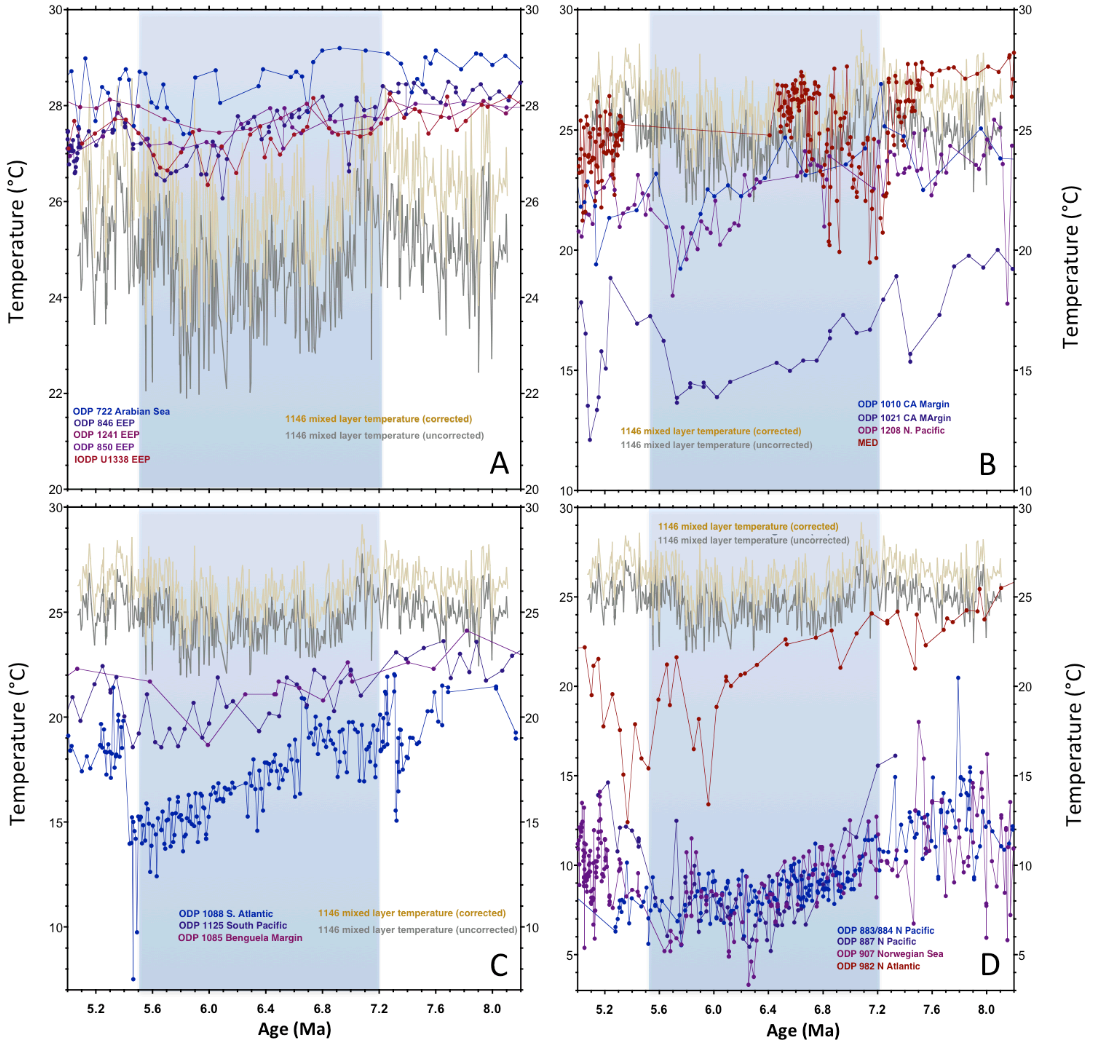

Supplementary Fig. 8. Comparison of published Uk'$_{37}$ SST records between 8.2 and 5 Ma to Site 1146 Mg/Ca derived mixed layer temperatures. **A:** tropics. **B:** Northern Hemisphere (30–50°N). **C:** Southern Hemisphere (30–50°S). **D:** Northern Hemisphere (>50°N). Note that the marked cooling registered in mid- and high-latitude regions between 7.2 and 5.3 Ma is also evident at Site 1146. The Uk'$_{37}$ SST data consistently use the calibration from ref. 22 and are based on the compilation in ref. 19. The Site 1146 Mg/Ca derived mixed layer temperatures are shown uncorrected (dark gray) and corrected (beige) using a modified calibration equation with a late Miocene Mg/Ca of seawater in the range of 4.5 mol/mol to adjust for potential differences between Miocene and modern Mg/Ca of seawater[17].

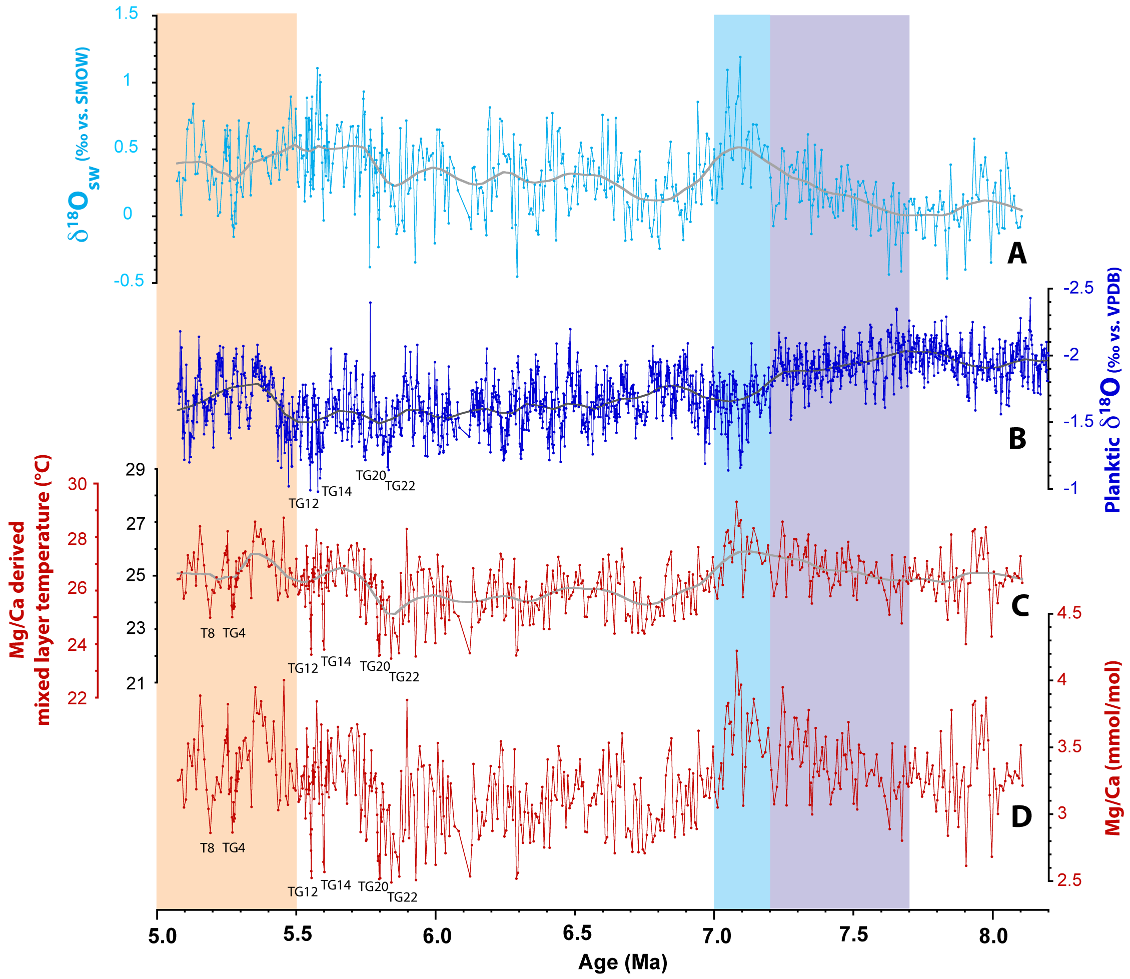

Supplementary Fig. 9. Reconstruction of mixed layer temperature and $\delta^{18}O_{sw}$ at ODP Site 1146. **A:** Oxygen isotope composition of sea water ($\delta^{18}O_{sw}$) estimated using the equation for *Orbulina universa* (LL – low light) from ref. 23. **B:** Planktic foraminiferal $\delta^{18}O$. **C:** Mixed layer temperature reconstruction with (red scale) and without (black scale) correction for secular changes in Mg/Ca of seawater. **D:** Planktic (*G. sacculifer*) Mg/Ca ratios. Smooth curves fitted using locally weighted least squared error (Lowess) method.

Supplementary Note 4: Factors influencing $\Delta\delta^{13}C_{planktic-benthic}$

The equilibration time for $\delta^{13}C$ in the mixed layer of the ocean, which is in the order of a decade, is sensitive to the DIC/CO_2 ratio. Thus, equilibration time is inversely correlated with atmospheric pCO_2. Model simulations[24] have shown that under low (e.g., Last Glacial Maximum) pCO_2, the disequilibrium between atmosphere/upper ocean and deep water is increased by >0.2 ‰ relative to pre-industrial values, whereas more rapid equilibration under high pCO_2 decreases isotopic disequilibrium (the surface/deep water $\delta^{13}C$ gradient). As a consequence, vertical $\delta^{13}C$ gradients in the ocean were lowered by up to 1 ‰ with respect to pre-industrial values during intervals of high pCO_2 (in the order of 500 ppm), which may have been reached during warm periods of the middle and late Miocene[25].

However, the interpretation of $\delta^{13}C$ gradients is additionally complicated by processes associated with changes in deep water ventilation (decreases/increases in benthic $\delta^{13}C$) and local surface productivity (removal of ^{12}C by primary producers). In particular, the gradient may be reduced, when benthic foraminiferal $\delta^{13}C$ increases due to processes in the source region of deep waters or variations in the mixing of deep water masses (in the case of the South China Sea: variations in the relative proportion of North Pacific Intermediate Water and southern component deep water). Nevertheless, planktic-benthic $\delta^{13}C$ gradients in the South China Sea reasonably reflect global atmospheric pCO_2 changes even for the last glacial cycles (Supplementary Fig. 10). The strong covariance of benthic and planktic $\delta^{13}C$ over the entire 4 Myr interval studied points to global reservoir effects as the driver of long-term $\delta^{13}C$ variability rather than changes in local circulation or productivity patterns.

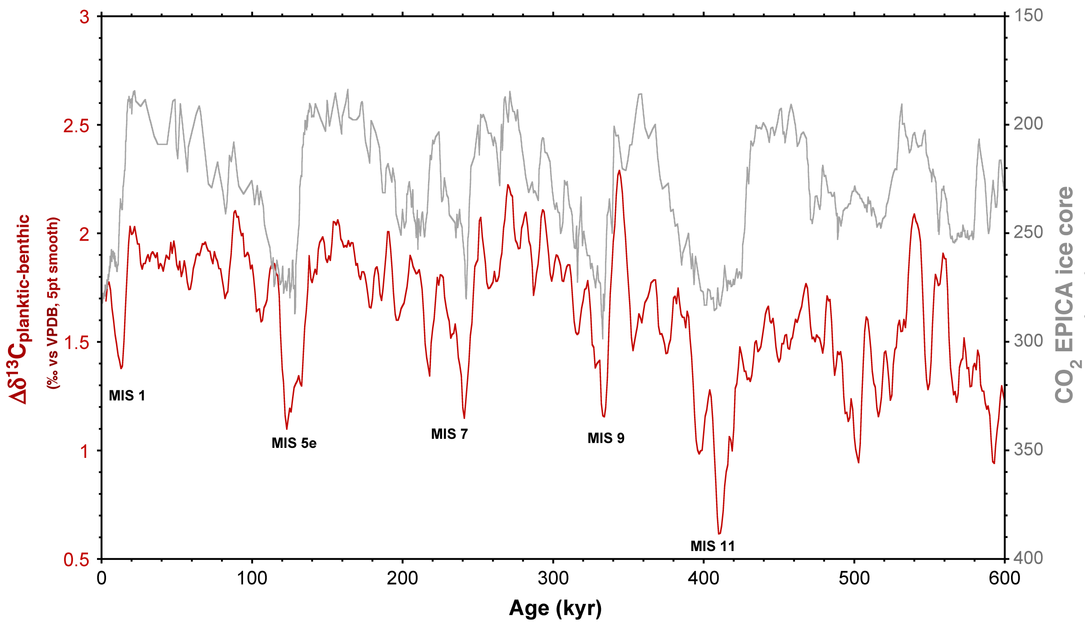

Supplementary Fig. 10. Gradient between planktic and benthic foraminiferal δ^{13}C ($\Delta\delta^{13}$C) in ODP Site 1146 (data from ref. 26 and unpublished) plotted vs. EPICA ice core pCO$_2$ between 600 and 0 ka (data from refs. 27–28). 5 pt smooth: 5 pt moving mean.

Supplementary references

1. Laskar, J., Robutel, P., Joutel, F., Gastineau, M., Correia, A. C. M., Levrard, B. A long-term numerical solution for the insolation quantities of the Earth. *Astron. Astrophys.* **428**, 261–285 (2004).
2. Holbourn, A. E., Kuhnt, W., Clemens, S., Prell, W., Andersen, N. Middle to late Miocene stepwise climate cooling: Evidence from a high-resolution deep water isotope curve spanning 8 million years. Paleoceanography 28, 688–699 (2013).
3. Van der Laan, E., Gaboardi, S., Hilgen, F. J., Lourens, L. J. Regional climate and glacial control on high-resolution oxygen isotope records from Ain el Beida (latest Miocene, northwest Morocco): A cyclostratigraphic analysis in the depth and time domain. *Paleoceanography* **20**, PA1001 (2005).

4. Van der Laan, E., Hilgen, F. J., Lourens, L. J., de Kaenel, E., Gaboardi, S., Iaccarinso, S. Astronomical forcing of Northwest African climate and glacial history during the late Messinian (6.5-5.5 Ma). *Palaeogeogr. Palaeoclim. Palaeoecol.* **313-314**, 107–126 (2012).
5. Paillard, D., Labeyrie, L., Yiou, P. Macintosh program performs time-series analysis. *EOS Trans. AGU* **77**, 379 (1996).
6. Torrence, C., Compo, G. P. A practical guide to wavelet analysis. *Bull. Am. Meteorol. Soc.* **79**, 61–78 (1998). URL: http://atoc.colorado.edu/ research/wavelets/.
7. Grinsted, A., Moore, J. C., Jevrejeva, S. Application of the cross wavelet transform and wavelet coherence to geophysical time series. *Nonlin. Processes Geophys.* **11**, 561–566 (2004).
8. Zeng, L., Wang, D., Chen, J., Wang, W., Chen, R. SCSPOD14, a South China Sea physical oceanographic dataset derived from in situ measurements during 1919–2014. Sci. Data **3**, 160029 (2016).
9. Broecker, W. S., Peng, T. -H. *Tracers in the Sea* (Lamont-Doherty Earth Observatory, 1982).
10. Stanley, S. M., Hardie, L. A. Secular oscillations in the carbonate mineralogy of reef-building and sediment-producing organisms driven by tectonically forced shifts in seawater chemistry. *Palaeogeogr. Palaeoclim. Palaeoecol.* **144**, 3-19 (1998).
11. Dickson, J. A. D. Fossil echinoderms as monitor of the Mg/Ca ratio of Phanerozoic oceans. *Science* **298**, 1222-1224 (2002).
12. Horita, J., Zimmermann, H., Holland, H. D. Chemical evolution of seawater during the Phanerozoic: implications from the record of marine evaporates. *Geochim. Cosmochim. Acta* **66**, 3733-3756 (2002).
13. Coggon, R. M., Teagle, D. A., Smith-Duque, C. E., Alt, J. C., Cooper, M. J. Reconstructing past seawater Mg/Ca and Sr/Ca from mid-ocean ridge flank calcium carbonate veins. *Science* **327**, 1114-1117 (2010).
14. Rausch, S., Böhm, F., Bach, W., Klügel, A., Eisenhauer, A. Calcium carbonate veins in ocean crust record a threefold increase of seawater Mg/Ca in the past 30 million years. *Earth Planet. Sci. Lett.* **362**, 215-224 (2013).
15. Lear, C. H., Coxall, H.K., Foster, G.L., Lunt, D.J., Mawbey, E.M., Rosenthal, Y., Sosdian, S. M., Thomas, E., Wilson, P.A. Neogene ice volume and ocean

temperatures: Insights from infaunal foraminiferal Mg/Ca paleothermometry, Paleoceanography **30**, 1437–1454 (2015).

16. Evans, D., Brierley, C., Raymo, M. E., Erez, J., Müller, W. Planktic foraminifera shell chemistry response to seawater chemistry: Pliocene–Pleistocene seawater Mg/Ca, temperature and sea level change. *Earth Planet. Sci. Lett* **438**, 139–148 (2016).
17. Lear, C. H., Elderfield, H., Wilson, P. A. Cenozoic deep-sea temperatures and global ice volumes from Mg/Ca in benthic foraminiferal calcite. *Science* **287**, 269–272 (2000).
18. Herbert, T. D., Lawrence, K. T., Tzanova, A., Peterson, L. C., Caballero-Gill, R., Kelly, C. S. Late Miocene global cooling and the rise of modern ecosystems. *Nat. Geosci.* **9**, 843–847 (2016).
19. Zweng, M. M., Reagan, J. R., Antonov, J. I., Locarnini, R. A., Mishonov, A. V., Boyer, T. P., Garcia, H. E., Baranova, O. K., Johnson, D. R., Seidov, D., Biddle, M. M. World Ocean Atlas 2013, Volume 2: Salinity. In *NOAA Atlas NESDIS, vol. 74* (eds Levitus, S., Mishonov, A.) 1–39 (NOAA, 2013).
20. Morimoto, M., Abe, O., Kayanne, H., Kurita, N., Matsumoto, E., Yoshida, N. Salinity records for the 1997-98 El Nino from Western Pacific corals. *Geophys. Res. Lett.* **29**, doi:10.1029/2001GL013521 (2002).
21. Liu, J., Song, X., Yuan, G., Sun, X., Yang, L. Stable isotopic compositions of precipitation in China, Tellus B: *Chemical and Physical Meteorology* **66**, 22567 (2014).
22. Müller, P. J., Kirst, G., Ruhland, G., Von Storch, I., Rosell-Melé, A. Calibration of the alkenone paleotemperature index Uk'37 based on core-tops from the eastern South Atlantic and the global ocean (60N-60S) *Geochem. Cosmochim. Acta* **62**, 1757–1772 (1998).
23. Bemis, B. E., Spero, H. J., Bijma, J., Lea, D. W. Reevaluation of the oxygen isotopic composition of planktonic foraminifera: Experimental results and revised paleotemperature equations. *Paleoceanography* **13**, 150–160 (1998).
24. Galbraith, E. D., Kwon, E. Y., Bianchi, D., Hain, M. P., Sarmiento, J. L. The impact of atmospheric pCO2 on carbon isotope ratios of the atmosphere and ocean. *Global Biogeochem. Cycles* **29**, 307–324 (2015).

25. Zhang, Y.G., Pagani, M., Liu, Z., Bohaty S.M., DeConto, R. A 40-million-year history of atmospheric CO2. *Phil. Trans. R. Soc. A* **371**, 20130096 (2013).
26. Clemens, S. C., Prell, W. L. Data report: Oxygen and carbon isotopes from Site 1146, northern South China Sea. In *Proc. ODP Scientific Results* **184**, (eds Prell, W. L., Wang, P., Blum, P., Rea, D. K., Clemens, S. C.) 1–8 (2003). [Online]. Available from World Wide Web: <http://www-odp.tamu.edu/publications/ 184_SR/ VOLUME/CHAPTERS/214.PDF>.
27. Lüthi, D., et al. EPICA Dome C Ice Core 800 kyr Carbon Dioxide Data. IGBP PAGES/World Data Center for Paleoclimatology Data Contribution Series # 2008-055. NOAA/NCDC Paleoclimatology Program, Boulder CO, USA (2008a).
28. Lüthi, D., Le Floch, M., Bereiter, B., Blunier, T., Barnola, J.-M., Siegenthaler, U., Raynaud, D., Jouzel, J., Fischer, H., Kawamura, K., Stocker, T.F. High-resolution carbon dioxide concentration record 650,000-800,000 years before present. *Nature* **453**, 379–382 (2008b).
